# Temporally and anatomically specific contributions of the human amygdala to threat and safety learning

**DOI:** 10.1073/pnas.2204066119

**Published:** 2022-06-21

**Authors:** Zhenfu Wen, Candace M. Raio, Edward F. Pace-Schott, Sara W. Lazar, Joseph E. LeDoux, Elizabeth A. Phelps, Mohammed R. Milad

**Affiliations:** ^a^Department of Psychiatry, New York University Grossman School of Medicine, New York, NY 10016;; ^b^Department of Psychiatry, Massachusetts General Hospital and Harvard Medical School, Charlestown, MA 02114;; ^c^Athinoula A. Martinos Center for Biomedical Imaging, Massachusetts General Hospital, Charlestown, MA 02129;; ^d^Center for Neural Science and Department of Psychology, New York University, New York, NY 10003;; ^e^Department of Child and Adolescent Psychiatry, New York University Grossman School of Medicine, New York, NY 10016;; ^f^Department of Psychology, Harvard University, Cambridge, MA 02138;; ^g^Neuroscience Institute, New York University Grossman School of Medicine, New York, NY 10016;; ^h^Center for Biomedical Imaging and Neuromodulation, Nathan Kline Institute for Psychiatric Research, Orangeburg, NY 10962

**Keywords:** amygdala, threat conditioning, fMRI

## Abstract

Neural plasticity in subareas of the rodent amygdala is widely known to be essential for Pavlovian threat conditioning and safety learning. However, less consistent results have been observed in human neuroimaging studies. Here, we identify and test three important factors that may contribute to these discrepancies: the temporal profile of amygdala response in threat conditioning, the anatomical specificity of amygdala responses during threat conditioning and safety learning, and insufficient power to identify these responses. We combined data across multiple studies using a well-validated human threat conditioning paradigm to examine amygdala involvement during threat conditioning and safety learning. In 601 humans, we show that two amygdala subregions tracked the conditioned stimulus with aversive shock during early conditioning while only one demonstrated delayed responding to a stimulus not paired with shock. Our findings identify cross-species similarities in temporal- and anatomical-specific amygdala contributions to threat and safety learning, affirm human amygdala involvement in associative learning and highlight important factors for future associative learning research in humans.

## Significance

Pavlovian threat learning is a primary translational model for understanding the brain systems that underlie anxiety and trauma-related psychopathology. The amygdala has traditionally played a central role in this important form of learning across species. However, recent human neuroimaging work has revealed inconsistent findings regarding the role of human amygdala in threat and safety learning. To address this discrepancy, we examined amygdala responses to threat-predictive cues in a large sample of human participants. We found robust evidence for amygdala responses during threat conditioning and, further, that these responses occurred in a temporally and anatomically specific manner. Our results reveal clear evidence of human amygdala involvement in associative learning and offer insight into why some neuroimaging work has yielded equivocal findings.

Learning associations between neutral and aversive stimuli is an evolutionarily adaptive process that occurs rapidly. Pavlovian threat conditioning paradigms are the primary translational model for this process in laboratory studies, during which a neutral stimulus comes to elicit a defensive response via its pairing with an aversive event. Studies in rodents have demonstrated the precise neural mechanisms within the amygdala that are essential for this associative learning (1–3). Studies in humans have also confirmed the general role of the amygdala in threat conditioning using lesion studies (4–6); yet neuroimaging studies have yielded less consistent results (7–14). This has led some to question the translational relevance of the well-established, cross-species role of the amygdala in threat conditioning (10, 14, 15).

Here, we argue that multiple factors might contribute to inconsistencies in the amygdala’s involvement in the human threat conditioning literature. One possibility is that most analytic approaches pay little attention to the temporal trajectory of amygdala responses during associative learning. More often than not, investigators average blood oxygenation level–dependent (BOLD) response signal across all conditioning trials during threat conditioning. Rodent studies, however, clearly indicate that the amygdala contributes to threat conditioning in a temporally specific manner, with the most reliable amygdala response occurring early in threat learning (16–18). A few prior neuroimaging studies also reported that amygdala BOLD signal was rapidly habituating during threat conditioning and a subsequent extinction retrieval test in small samples (7 ∼ 18 subjects) (8, 11, 19). Thus, averaging BOLD signal across an entire learning phase could result in a weak or minimal amygdala signal detection. Another possibility is the lack of focus on the amygdala’s anatomical specificity, given that it is composed of functionally distinct subregions in rodents (1, 3, 20, 21) and humans (22, 23). Averaging BOLD response across these multiple subnuclei could further weaken potential detection of the amygdala’s contribution to threat conditioning. A third possibility is insufficient power to detect subtle, time-dependent, and anatomically specific amygdala responding during threat conditioning. Capturing the amygdala’s phasic and trial-dependent signal using BOLD imaging could be complicated by: 1) a very low spontaneous (baseline) firing rate within amygdala neurons; and 2) conditioning-induced neural plasticity in the rodent amygdala causing minimal increase in the overall neural firing rate (averaging about 0.4 to 1 Hz) (18, 21). Therefore, a relatively large sample might be required to robustly detect amygdala BOLD response to learned threat. Here, we examined these possibilities by combining data from 601 participants across multiple threat conditioning studies, yielding a large sample that was adequately powered to detect the temporal changes of BOLD responses in subareas of the amygdala during threat and safety learning. All participants underwent an identical threat conditioning paradigm that lasted ∼800 s while in functional MRI (fMRI) scanners (*SI Appendix*, Fig. S1 and *Methods*). Subjects were presented with three different colored lights (red, blue, and yellow) as the conditioned stimuli (CS). During the first block of the conditioning, one conditioned stimulus (CS+1; e.g., blue light) was presented for 8 trials; 5 of the 8 presentations coterminated with a mild electric shock (unconditioned stimulus [US], 62.5% reinforcement rate). During the second conditioning block, another light color (e.g., yellow) was presented for 8 trials with a 62.5% reinforcement rate (CS+2). Intermingled with the CS+s were 16 trials of a different color (e.g., red) that was never paired with shock (CS).

## Results

**Fig. 1.**
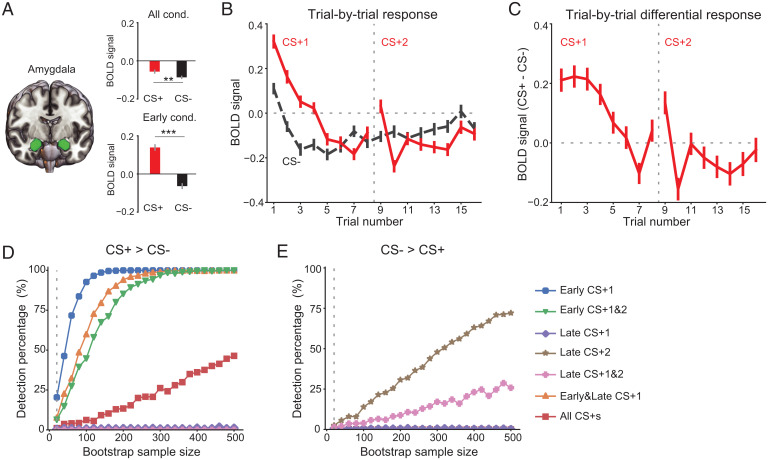
Temporal-specific amygdala response during threat conditioning. (*A*) Amygdala BOLD response to CS+ was significantly higher relative to CS−. The tests were conducted either with *z*-scored BOLD signal from all conditioning trials (*t*_600_ = 2.68, *P* = 0.008, Cohen’s *d* = 0.12) or early conditioning trials (first four trials of CS+1 and CS−, *t*_600_ = 10.11, *P* < 0.001, Cohen’s *d* = 0.51). (*B* and *C*) Trial-by-trial amygdala BOLD response to each CS type (*B*) and differential response between CS+ and CS− (*C*). The vertical dashed lines mark the separation of the first and the second CS block. Error bars indicate SEs across participants. (*D* and *E*) Percentage of detected significant activation difference (CS+ vs. CS−) as a function of sample size, separately for CS+ > CS− (*D*) and CS− > CS+ (*E*). The bootstrap resampling procedure was repeated 1,000 times for each sample size (from 20 to 500, step size: 20). ***P < 0.001; **P < 0.01.

We first used the same analytic strategy as most neuroimaging threat conditioning studies by evaluating the whole amygdala BOLD signal across the experiment in 601 participants. We compared the mean BOLD response of all 16 CS+ trials to all 16 CS− trials across threat conditioning. This analysis revealed a significantly higher amygdala BOLD response to the CS+ compared to the CS− (*t*_600_ = 2.68, *P* = 0.008, Fig. 1*A*), a finding that supports the majority of fMRI studies in this domain. Despite this statistically significant result, we note that the effect size is fairly small (Cohen’s *d* = 0.12). We reasoned that this observed small effect size could be due to loss of the transient signal as a consequence of averaging the BOLD responses in the amygdala across all conditioning trials. Given that the rodent amygdala signals the CS+ peak within the first few conditioning trials (16–18), we focused our next analysis on the BOLD response within the first 4 CS+ trials and the corresponding 4 CS− trials. This analysis revealed a robust amygdala BOLD response to the CS+ compared to the CS− (*t*_600_ = 10.11, *P* < 0.001, Fig. 1*A*) with a medium effect size (Cohen’s *d* = 0.51). We then examined trial-by-trial amygdala activation across all 32 CS trials (Fig. 1 *B* and *C*). Consistent with findings in rodents, the amygdala BOLD response was stronger in the CS+ relative to the CS− early in the threat acquisition phase and showed rapid habituation thereafter.

Considering the dynamic nature of the amygdala BOLD signal, we next reasoned that including different subsets of trials and different sample sizes could influence the statistical results. To test this, we conducted bootstrap analyses (with bootstrap sample sizes range from 20 to 500) to examine how different analytic strategies might influence the likelihood of detecting significant amygdala BOLD responses (*Materials and Methods*). In our paradigm, we found that obtaining consistently significant BOLD response to the CS+ (>75% detection rate) requires ∼80 subjects when using trials from the early phase of threat conditioning (Fig. 1*D*). Using early trials of both CS+s or all trials from the first CS+ also led to relatively robust detections of amygdala response, which required less than 200 subjects to obtain a 75% detection rate. However, when using this bootstrapping approach on all conditioning trials, our ability to detect robust activation was diminished—even with 500 subjects. We also examined the effect size using different analytic strategies and sample sizes (*SI Appendix*, Fig. S2). Consistent with the above analysis, using trials from early threat conditioning led to a medium effect size (Cohen’s *d* > 0.5), which was much stronger than using all trials (Cohen’s *d* < 0.15). Note that the 95% confidence intervals are of wide range with bootstrap sample size smaller than 200 (as in the case of most human threat conditioning studies). This large sample variability may partially explain the inconsistencies across studies in detecting the amygdala BOLD response during threat conditioning. Overall, these results point to the importance of the temporally dependent nature of amygdala involvement in threat conditioning (as measured by the BOLD response).

To control for any potential confounds of shock delivery into the BOLD signal, we conducted additional analyses focusing only on the BOLD response from the six unreinforced CS+ trials. These analyses revealed a consistent increase in amygdala BOLD signal to the CS+ vs. CS− in early threat conditioning (*SI Appendix*, Fig. S3). As can be noted from *SI Appendix*, Fig. S3, our results make clear the need for an even larger sample size to detect changes in amygdala BOLD signal to CS+ if fewer trials are selected for the analyses. In addition to detecting early CS+ vs. CS− BOLD signal in the whole amygdala, our trial-by-trial analyses in late threat conditioning show higher BOLD response to the CS− relative to the CS+ (Fig. 1*C*). To reliably observe this finding, however, a large sample size is needed (Fig. 1*E*). Specifically, the highest detection rate for CS− > CS+ was around 70%, which was obtained when late trials of the second CS block of 500 subjects were used. If only unreinforced CS+ trials were used, the detection rate decreased to 30% (*SI Appendix*, Fig. S3).

To test for the durability of amygdala responsivity to the CS+ postconditioning, we examined the amygdala BOLD responses during the early phase of extinction learning (first four trials immediately after threat conditioning) and early extinction memory recall (first 4 trial 24 h after threat conditioning). These analyses revealed that amygdala responses were stronger in the CS+ relative to the CS− at the very beginning of each of these two phases, then habituated quickly (*SI Appendix*, Fig. S4). The results obtained from these analyses are consistent with rodent studies showing CS-evoked amygdala activity lasting days to weeks after threat conditioning (24, 25).

**Fig. 2.**
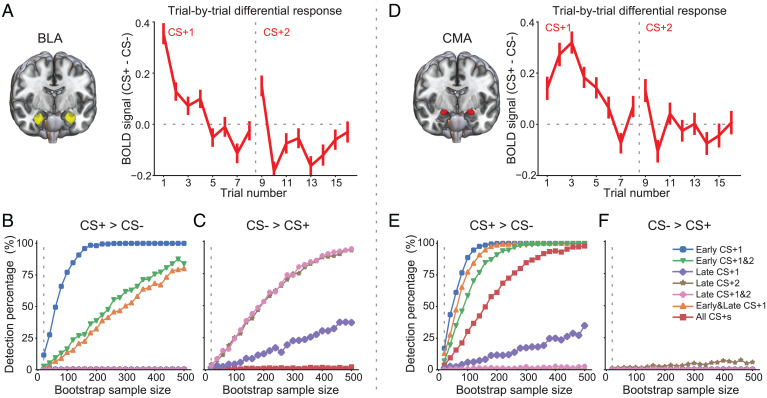
Distinct activation patterns of the two amygdala subdivisions. (*A*) Trial-by-trial differential BOLD response for BLA. The *z*-scored BOLD signal difference between CS+ and CS− is shown for each trial. The vertical dashed line marks the separation of the first and the second CS block. Error bars indicate SEs across participants. (*B* and *C*) Percentage of detected significant BOLD difference (CS+ vs. CS−) as a function of sample size, separately for CS+ > CS− (*B*) and CS− > CS− (*C*). The bootstrap resampling procedure was repeated 1,000 times for each sample size (from 20 to 500, step size: 20). (*D–F*) Similar as in *A–C*, except for CMA.

We next examined the anatomical specificity of the BOLD signal associated with conditioned stimuli. We focused on two broad regions—the basolateral (BLA) and centromedial (CMA) amygdala, which have been implicated in distinct contributions to learning and behavioral expression of threat associations in rodent studies (3, 20, 21). Although it is not currently possible to dissect the human amygdala with such fine anatomical resolution using MRI, anatomical (22) and functional (26, 27) data in humans have identified ventral and dorsal amygdala regions that seem to correspond to the BLA and CMA in rodents. For the purposes of this study, we therefore refer to these ventral and dorsal subregions as BLA and CMA, respectively, while keeping these caveats in mind. We first examined trial-by-trial BOLD responses of BLA and CMA. Within putative BLA, we observed initially higher BOLD signal to the CS+ vs. CS− that quickly habituated across the conditioning phase, as in the rodent amygdala (Fig. 2 *A* and *B*). Moreover, by the late stage of conditioning, we observed stronger BLA BOLD signal to the CS− relative to CS+ (Fig. 2 *A* and *C*), suggesting the formation of a “CS− no US” association. Within the dorsal region (CMA), we observed a peak increase in BOLD signal by the third trial of threat conditioning that diminished later in the conditioning phase (Fig. 2 *D*–*F*). As in the rodent amygdala, these findings suggest the development of a learning signal pertaining to the formation of a CS–US association. No significant change in BOLD signal was observed late in the threat conditioning phase in CMA.

**Fig. 3.**
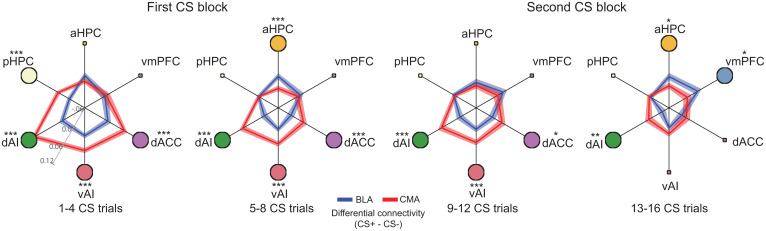
Distinct functional connectivity patterns for the two amygdala subdivisions. The differential connectivity (connectivity during CS+ processing minus connectivity during CS− processing) between BLA/CMA and brain regions central to associative learning from early (*Left*) to late (*Right*) conditioning phase. Each panel represents connectivity estimated with four trials of each CS type. Brain regions showing significant connectivity differences between BLA and CMA (*P*_FDR_ < 0.05) are highlighted as large circles around the periphery. ***P < 0.001; **P < 0.01; *P < 0.05.

To further test the functional distinction between BLA and CMA, we conducted stimulus-specific (CS+ vs. CS−) functional connectivity analyses (28, 29). We focused on the connectivity between BLA/CMA and brain regions that are central to threat conditioning and extinction (10, 30–33), including ventromedial prefrontal cortex (vmPFC), hippocampus (anterior [aHPC] and posterior [pHPC] parts), dorsal anterior cingulate cortex (dACC), and anterior insula (dorsal [dAI] and ventral [vAI] part). BLA and CMA demonstrated distinct functional connectivity patterns with these regions (Fig. 3). Specifically, BLA revealed stronger functional connectivity with aHPC and vmPFC during late stages of threat conditioning. In contrast, CMA exhibited stronger functional connectivity with dAI, vAI, pHPC, and dACC compared to BLA (false discovery rate [FDR] correction, *P*_FDR_ < 0.05), particularly at the early stages of threat conditioning. While the significant differences in functional connectivity subsided during late threat conditioning between some of the nodes, functional connectivity patterns between CMA, the insular cortex, and the dACC remained significant throughout most of the threat conditioning phase.

Finally, we conducted additional analyses to examine potential functional connectivity differences between BLA and CMA and previously defined neural networks (34) known to be critical for conscious awareness, declarative memory, and attention processes in humans. The two amygdala subdivisions exhibited prominent differences in functional connectivity patterns with these distributed neural systems (*SI Appendix*, Fig. S5). Thus, functional connectivity changes between BLA and CMA and some neural systems showed transient statistical significance, echoing the temporal changes of BOLD responses, while some others like ventral attention and frontoparietal control networks, showed consistent differences in functional connectivity throughout most of the conditioning phase.

## Discussion

Although there are apparent inconsistencies in finding robust amygdala activation during threat conditioning paradigms in humans (7–14), using a well-powered dataset we show a clear temporal profile for amygdala BOLD responses in threat conditioning. We establish cross-species translation within specific regions of the human amygdala that contribute to signaling the acquisition of threat conditioning as well as signaling the safety of the conditioned stimulus not paired with aversive stimuli.

We identified robust amygdala BOLD responses to conditioned stimuli paired with an aversive event (CS+), particularly in the early stage of the threat conditioning. The elevated response to CS+ rapidly habituated after a few trials. This suggests that inconsistencies in amygdala responses across studies may be because the majority of human neuroimaging studies assessing threat conditioning have not adequately considered the temporal trajectory of associative threat learning identified in animal models. It is well documented in rodent studies that the amygdala contributes to threat conditioning in a temporally specific manner, with the most reliable amygdala response occurring early in learning. For example, studies in rodents have shown tone responses signaling the CS+ peak within the first three to four conditioning trials; these responses were no longer measurable within the subsequent five to six trials (16–18). However, fMRI studies typically average BOLD responses to the CS+ and CS− across all conditioning trials. This is done to reliably estimate the BOLD response, given the relatively low number of subjects recruited into a single study. Given the evidence from the animal literature, it should not be surprising that amygdala activation is not consistently observed when the BOLD response is averaged across a large number of trials during a long conditioning phase while still applying stringent statistical criteria in relatively small samples. This view is supported by our results showing that the effect size in detecting the amygdala BOLD response increased when we focused on the early stage rather than the whole threat conditioning.

We demonstrated that the BLA and CMA exhibit distinct activation patterns during the acquisition of threat conditioning. Both BLA and CMA exhibit stronger responses to CS+ than CS− in early conditioning, but only the BLA shows higher response to CS− than CS+ in late conditioning. This functional heterogeneity of human amygdala subregions is consistent with findings reported in human resting-state (23, 27) and task-based (26, 35) fMRI studies, as well as in rodent studies (3, 20, 21). In the rodent amygdala, the lateral nucleus of the BLA is often associated with the acquisition of the CS–US association, whereas the basal nucleus exhibits mixed populations of neurons; some signal the acquisition of the CS–US association, while others contribute to extinction learning (3). The central nucleus of the CMA region is thought to be involved in response expression, but also has mixed populations that contribute to learning and extinction (20). The development of the differential BOLD signal of BLA in late acquisition is consistent with data indicating that extinction-learning–induced neural firing in subsets of amygdala neurons develop in the later trials during safety learning (36, 37), support the role of the human amygdala in associative learning related to safety (CS− is associated with no shock) (14, 38). Furthermore, we observed a distinct pattern of functional connectivity between BLA/CMA and other brain regions that are central to threat conditioning and extinction (or the “threat network”). Stronger connections between BLA and vmPFC and aHPC in late conditioning is consistent with rodent studies showing a role of the BLA in safety learning via its connections with the hippocampus and mPFC and a prominent role of the CMA in signaling threat conditioning via its connections with the dACC and insular cortex (36, 39). Beyond the threat network, we observed distinct functional connectivity between BLA/CMA and multiple neural systems, suggesting that conditioning-induced neural plasticity within the amygdala may interact with multiple subcortical and cortical areas involved in conscious awareness of threat and fear (40–42).

Although we observed some consistency between fMRI data and rodent neurophysiological measures, it does not necessarily mean that the fMRI data we report fully represent the complexity of different neuronal subtypes involved in encoding threat conditioning within the amygdala. Recent rodent studies using advanced imaging methods have revealed multiple cell types within the amygdala (43), such that some neurons exhibit up- or down-regulation of CS-evoked responses, while others remain responsive across multiple trials during threat conditioning. And given that the BOLD signal reflects global changes among thousands of neurons, the dynamic changes occurring among subtypes of single neurons within the amygdala might be underestimated/undetectable in fMRI studies.

It is possible that the strength of the amygdala BOLD signal and the temporal profile observed in our study might differ from other studies, given that stimulus types, temporal parameters, and reinforcement rate differ between paradigms. These paradigm differences may partially explain some inconsistencies in human threat conditioning studies (10, 14), especially given that only a subset of studies consider the temporal characteristics of this learning when assessing amygdala BOLD responses (7, 8, 11, 44). We showed here that detection of a subtle brain signal requires a large sample size. Another important factor that will affect the statistical power is the amount of data acquired from each subject. Recent precision functional mapping studies have shown that large amounts of fMRI data from individuals can improve the signal-to-noise ratio and allow for individual-specific functional neuroanatomy (45, 46), such as individualized amygdala subdivisions (23). These precision functional mapping approaches can be applied in future studies to facilitate detection of the temporally and anatomically specific amygdala responses during threat conditioning.

In sum, our findings point to the necessity of carefully examining and modeling temporal dynamics (47), of making anatomical distinctions, and to a critical role for sample size when evaluating the role of the human amygdala in associative learning and memory using BOLD imaging.

## Materials and Methods

### Participants.

Neuroimaging data from a total of 601 participants (age: 31.3 ± 12.4; 391 female, 210 male) across multiple studies were included in the analyses. Among these participants, 395 were healthy controls, 114 were diagnosed with posttraumatic stress disorder, and 92 were diagnosed with anxiety disorders. Impact of diagnoses on amygdala BOLD responses was not considered in any of the analyses as that is not a primary interest or objective for this study. Results from different subsets of the participants included in the analyses had been previously published with different foci and analytic strategies (48–52). Among participant inclusion criteria were: 18 to 65 y old, proficient in English, right-handed, and normal or corrected-to-normal vision. The exclusion criteria included: history of seizures or significant head trauma, current substance abuse or dependence, metal implants, pregnancy, breastfeeding, or positive urine toxicology screen for drugs of abuse. All participants were recruited at the Massachusetts General Hospital. All procedures were approved by the Partners HealthCare Institutional Review Board of the Massachusetts General Hospital. All participants provided written informed consent before they participated in the study.

### Experimental Design.

All participants underwent an identical threat conditioning paradigm (*SI Appendix*, Fig. S1) while BOLD responses were assessed. Before the experiment, electrodes were attached to the index finger and middle finger of the participant’s right hand for shock delivery, and participants were instructed to select the level of electric shock to be used in the experiment, so that the shock level was highly annoying but nonpainful. The experimental paradigm started with a habituation stage during which each of the paradigm images were presented to the participant once, without any electrical stimulation. During threat conditioning, subjects were presented with three different colored lights (red, blue, and yellow) within a room image (context) as the CS. The components of a trial are shown in *SI Appendix*, Fig. S1*A*. Specifically, each trial started with a 3-s presentation of the context image, followed by a 6-s presentation of the CS. In a CS+ trial, the CS coterminated with a 0.5-s electric shock. While in a CS− trial, the CS presentation was never followed by a shock. The intertrial interval (fixation image) was 15 s on average (range: 12 ∼ 18 s). The conditioning phase was divided into two blocks. During the first block, one CS+ (e.g., blue light, CS+1) was presented for 8 trials, with 5 of the 8 presentations coterminated with a mild electric shock (62.5% reinforcement rate). Intermingled with this CS+1 were 8 trials of a different color (e.g., red) that were never paired with shock (CS−). During the second block, a third light color (e.g., yellow) was presented for 8 trials with a 62.5% reinforcement rate (CS+2). An additional 8 CS− trial presentations were intermingled with this CS+2. In total, the conditioning phase consisted of 32 trials, including 16 CS− trials, 8 CS+1 trials during the first block, and another 8 CS+2 trials during the second block. The order of trials was pseudorandom. The colored lights used as CS+s and CS− were counterbalanced across participants.

### Image Acquisition and Preprocessing.

Neuroimaging data were acquired using three different MRI settings. Data from 119 participants were acquired in a Trio 3.0 Tesla whole-body MRI scanner (Siemens Medical Systems) using an 8-channel head coil. Functional data were acquired using a T2*-weighted echo-planar imaging (EPI) pulse sequence (repetition time [TR]: 3.0 s, echo time [TE]: 30 ms, slice number: 45, voxel size: 3 × 3 × 3 mm). Data from 386 participants were acquired in the same Trio 3.0 Tesla MRI scanner using a 32-channel head coil. Functional data were acquired using a T2*-weighted EPI pulse sequence (TR: 2.56 s, TE: 30 ms, slice number: 48, voxel size: 3 × 3 × 3 mm). Imaging data from 96 participants were acquired on a Siemen’s Prisma 3.0T equipped with a 32-channel head coil. Functional data were acquired using a T2*-weighted EPI pulse sequence (TR: 3.0 s, TE: 30 ms, slice number: 48, voxel size: 2.5 × 2.5 × 2.5 mm). High-resolution anatomical images were acquired for image registration.

Preprocessing was performed using the default pipeline of fMRIPrep 20.0.2—a standard toolbox for automatic fMRI data preprocessing (53). Functional images were corrected for slice timing, realigned, coregistered with the structural image, normalized into the Montreal Neurological Institute (MNI) space and smoothed with a 6-mm full-width half-maximum Gaussian kernel.

### Regions of Interest.

The amygdala mask was obtained from the Harvard–Oxford subcortical probabilistic atlas. A 50% probability threshold was applied to obtain a high anatomical specificity of the bilateral amygdala. For subregion analyses, the CMA and the BLA masks were created using the cytoarchitectonically defined probabilistic maps via the SPM Anatomy Toolbox (54). For connectivity analyses, the vmPFC and dACC masks were created using Neurosynth (55). We searched the keyword “conditioning” and identified the following peak coordinates: vmPFC (MNI_xyz_ = −2, 46, −10) and dACC (MNI_xyz_ = 0, 14, 28). An 8-mm sphere was created for each coordinate. For aHPC and pHPC, the Harvard–Oxford subcortical probabilistic atlas was used (50% threshold). The aHPC and pHPC were separated based on the location of the uncal apex in the MNI space (i.e., *Y* = −21 mm). The dAI and vAI masks were based on a functionally defined mask (56).

### Activation Analyses.

For the trial-by-trial BOLD response estimation, we used the least-squares-all-based generalized linear model (57) implemented via the Nistats 0.0.1rc toolbox. The model included a regressor for each of the CS presentations (32 regressors in total) and a regressor for the context presentation. Each regressor was modeled by convolving the onset of the stimulus with the two-gamma canonical hemodynamic response function using a duration of 6 s (CS) or 3 s (context). Other regressors, including the six motion parameters, high-pass temporal filtering (128 s) terms, and polynomial drift were included in the model. The volumes with framewise displacement larger than 0.9 were flagged as outliers and censored from parameter estimation (58). A first-order autoregressive model was used to account for the temporal structure of the noise. The contrast map for each trial was computed using a *t* test and *z* scored to assure standardized results that are independent of the number of observations. The *z*-scored BOLD response for each trial was averaged across voxels in the amygdala, BLA, or CMA to represent the BOLD response of each, respectively. These regional signals were used to compare BOLD responses during CS+ trials to CS− trials at the group level.

Considering the dynamic nature of the amygdala activity, we examined how different analytic strategies influence the statistical results. We separately divided the first and the second block into early and late phase, with each phase consisting of 4 trials of each CS type (*SI Appendix*, Fig. S1*B*). We tested seven different combinations of trials for significant tests, including: 1) all CS+s, which included 16 CS+ trials (8 CS+1, 8 CS+2) and 16 CS− trials; 2) early CS+1, which included trials from the early phase of the first CS block (4 CS+1, 4 CS−); 3) early CS+1 and 2, which included trials from the early phase of both the first and the second CS blocks (4 CS+1, 4 CS+2, 8 CS−); 4) late CS+1, which included trials from the late phase of the first CS block (4 CS+1, 4 CS−); 5) late CS+2, which included trials from the late phase of the second CS block (4 CS+2, 4 CS−); 6) late CS+1 and 2, which included trials from the late phases of both the first and the second CS blocks (4 CS+1, 4CS+2, 8 CS−); 7) early and late CS+1, which included trials from both the early and the late phases of the first CS block (8 CS+1, 8 CS−). For each analytic strategy, the *z*-scored BOLD signals of the corresponding trials were separately averaged for CS+ and CS− and then input to a paired *t* test to test for group-level activation difference (CS+ vs. CS−).

We evaluated the influence of sample size on the statistical results by using bootstrap analysis. The analysis included the following steps: 1) *n* participants were sampled from the total set (i.e., all 601 participants) with replacement; 2) two-tailed paired *t* test was used to compare CS+ vs. CS−, and a significant activation difference was thought to be detected if *P* < 0.01; 3) steps 1 and 2 were repeated 1,000 times to calculate the percentage of detected significant activation difference for CS+ > CS− and CS− > CS+ separately. The above procedure was repeated with *n* started from 20 to 500 (step size: 20) and for each analytic strategy.

### Connectivity Analyses.

We estimated stimulus-specific connectivity using a recently proposed cofluctuation time series method (28, 29). The method consisted of the following steps: 1) Let *z_i_* = [*z_i_*(1),…, *z_i_*(*T*)] and *z_j_* = [*z_j_*(1),…, *z_j_*(*T*)] be the *z*-scored time series of two regions/voxels *i* and *j* (a total of *T* volumes). We first calculated the componentwise product between *z_i_* and *z_j_*, to get a time series *c_ij_* = [*c_ij_*(1),…, *c_ij_*(*T*)] = [*z_i_*(1)*z_j_ (1),…, *z_i_*(*T*)*z_j_(*T*)]. 2) We constructed a design matrix in a similar way as in the activation analysis. Specifically, we divided the conditioning phase into twostages (4 CS+ and 4 CS− for each stage) and constructed two task regressors for each stage (one for CS+, one for CS−), by convolving the onset of the stimulus with the two-gamma canonical hemodynamic response function. The context presentations were modeled as one regressor. 3) The design matrix was input to a general linear model with *c_ij_* as the dependent variable and estimated using a first-order autoregressive model. 4) Stimulus-specific functional connectivity between *i* and *j* was then computed as contrast of parameters (CS+ vs. CS− at each stage here) and further *z* scored for group-level analysis.

We extracted mean time series of BLA/CMA, and estimated the strength of stimulus-specific connectivity between BLA/CMA and every voxel from other regions (i.e., vmPFC, dACC, aHPC, pHPC, vAI, and dAI). The mean connectivity strength across voxels from each region was used for significance testing (two-tailed paired *t* test, BLA vs. CMA). We also estimated the connectivity strength with BLA/CMA at a whole-brain level. Based on prior studies (34, 59, 60), the whole brain was divided into nine brain networks: visual (VIS), subcortical (SUB), somatomotor (SMN), ventral attention (VAN), limbic (LIM), dorsal attention (DAN), default mode (DMN), frontoparietal control (CON), and cerebellum (CBN) networks. Connectivity strength of voxels from a network was averaged into a single value to represent the connectivity strength between this network and BLA or CMA.

### Statistical Analyses.

Two-tailed paired *t* test was used to test the BOLD signal differences between CS+ and CS− and to test the connectivity pattern differences between CMA and BLA. The FDR method was used for multiple comparison correction. Effect size was calculated using Cohen’s *d*.

## Supplementary Material

Supplementary File

## Data Availability

Anonymized data and analysis code associated with this work are available at Open Science Framework (OSF) (https://osf.io/srvx7) (61).
