# Supplementary material for: Temporally and anatomically specific contributions of the human amygdala to threat and safety learning
Source: Proc Natl Acad Sci U S A. 2022 Jun 21;119(26):e2204066119. doi: 10.1073/pnas.2204066119 (PMC9245701; doi:10.1073/pnas.2204066119)
Supplement: Supplementary File [file pnas.2204066119.sapp.pdf]

PNAS

Supplementary Information for
Temporally and anatomically specific contributions of the human amygdala to threat and safety learning

Zhenfu Wen, Candace M. Raio, Edward F. Pace-Schott, Sara W. Lazar, Joseph E. LeDoux, Elizabeth A. Phelps, and Mohammed R. Milad

Joseph E. LeDoux
Email: jel1@nyu.edu
or
Mohammed R. Milad
Email: mohammed.milad@nyulangone.org

This PDF file includes:

Supplementary text
Figures S1 to S5
SI References

Supplementary Information Text

SI Results

Amygdala responses during extinction learning and extinction memory recall. After threat conditioning, most of these 601 participants also underwent extinction learning phase (immediately after threat conditioning) and an extinction memory recall phase (24 hours later) (see (1, 2) for more details). During extinction learning, one of the CS+s and the CS- were repeatedly presented with no shock. During extinction learning, all CS+s and the CS- were presented with no shock. We further examined the CS-evoked amygdala BOLD responses in early stage (the first 4 trials) of each phase (**Fig. S4**). During extinction learning (N = 594), amygdala BOLD responses were significantly higher to the CS+ relative to the CS- in the first trial (t_{593} = 4.25, P < 0.001), but then habituated quickly (the second trial: t_{593} = 2.11, P = 0.35). Similar patterns were observed when we separately examined BLA (the first trial: t_{593} = 2.84, P = 0.005, the second trial: t_{593} = 1.61, P = 0.11) and CMA (the first trial: t_{593} = 3.88, P < 0.001, the second trial: t_{593} = 2.26, P = 0.024). During extinction memory recall (N = 574), we averaged BOLD responses to the extinguished CS+ and the unextinguished CS+, since these two CS+s exhibited comparable responses (the first trial amygdala response: t_{573} = -0.59, P = 0.56). The amygdala BOLD activations were stronger to the CS+ relative to the CS- in the first trial (t_{573} = 6.41, P < 0.001), but then habituated quickly (the second trial: t_{573} = 1.33, P = 0.19). Similar patterns were observed in BLA (the first trial: t_{573} = 2.83, P = 0.005, the second trial: t_{573} = -0.22, P = 0.82) and CMA (the first trial: t_{573} = 8.82, P < 0.001, the second trial: t_{573} = 1.72, P = 0.09).

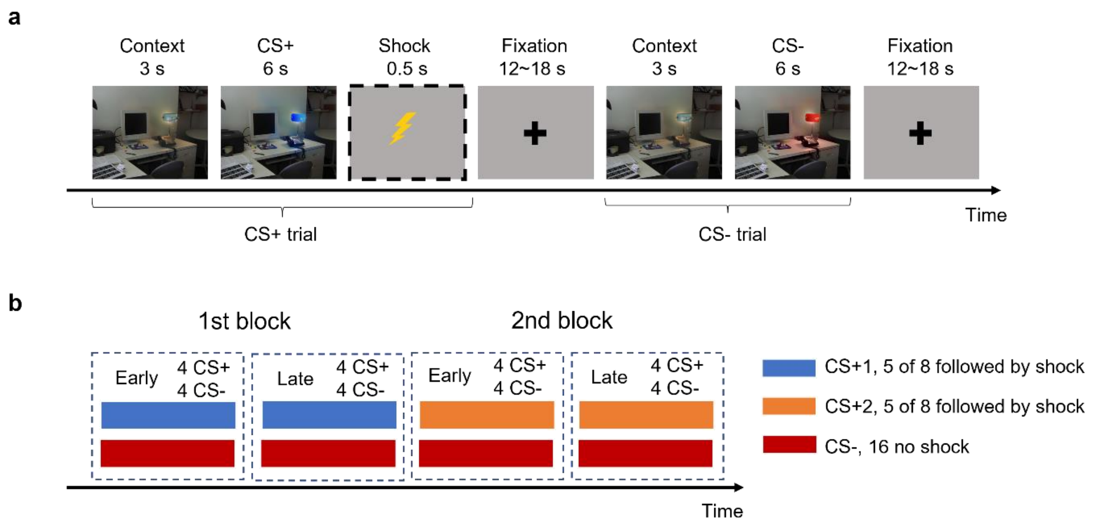

Fig. S1. Experimental design. a. Structure of CS+ and CS- trial. Each trial starts with a 3 s presentation of the context image (i.e., computer room here), followed by a 6 s presentation of the CS (colored light). A CS+ trial may be followed by a 0.5 s electric shock, while a CS- trial is never followed by a shock. The inter-trial interval (fixation) lasts 15 s on average (12~18 s). **b.** The overall structure of the threat conditioning experiment. Three different colored lights (blue, red, yellow) are presented. One color (e.g., blue) is presented for 8 trials, with mild 0.5 s electric shock delivered at the immediate offset of 5 out of 8 light presentations (62.5% reinforcement rate; conditioned stimulus [CS+1]). Intermingled with this CS+1 is 8 trials of a different light color that is never paired with the shock (CS-, e.g., red). The total 16 CS trials represent 1st CS block. During the 2nd block of the conditioning phase, a third light color (e.g., yellow) is paired with the shock (CS+2, 62.5% reinforcement rate, 8 trials). Additional 8 CS- trials are also intermingled with CS+2. Therefore, the experiment consists of 32 CS trials, including 16 CS- trials, 8 CS+1 trials during the first block, and another 8 CS+2 trials during the second block.

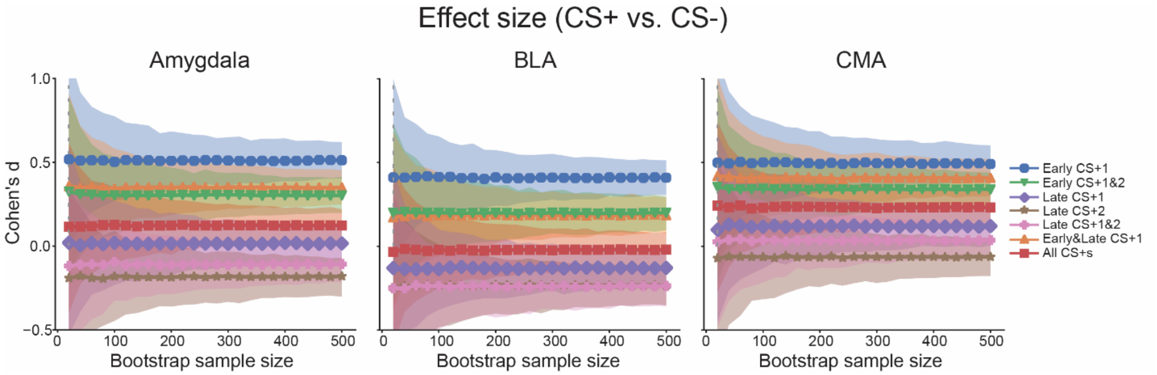

Fig. S2. Effect size as a function of sample size. The bootstrap resampling procedure was repeated 1000 times for each sample size (started from 20 to 500, step size: 20). Cohen's d was estimated for each resampling to compare CS+ and CS- using different subsets of trials. Shaded areas represent the 95% confidence interval estimated by the bootstrapping procedure.

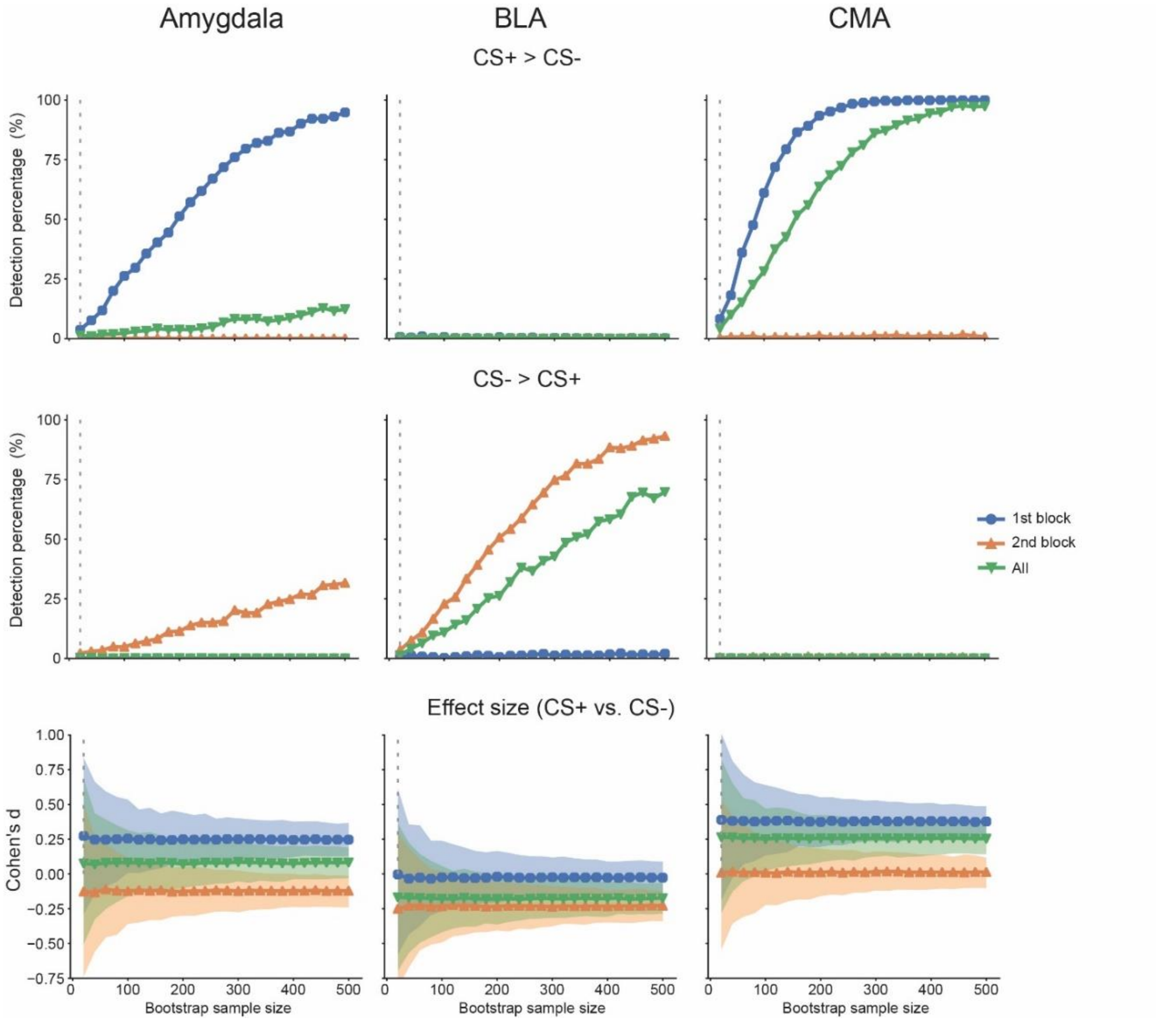

Fig. S3. Bootstrap analyses based on the unreinforced CS+ trials. There were 6 unreinforced CS+ trials, with 3 trials from the 1st CS block, 3 trials from the 2nd CS block. We tested 3 analytic strategies: only trials from the 1st block, only trials from the 2nd block, all 6 trials for CS+ vs. CS- comparison. With large sample size, we could detect stronger activation to the CS+ relative to the CS- in amygdala and CMA (first row), stronger activation to the CS- relative to the CS+ in BLA (second row). Effect sizes for CS+ vs. CS- are show in the third row. Shaded areas represent the 95% confidence interval estimated by the bootstrapping procedure.

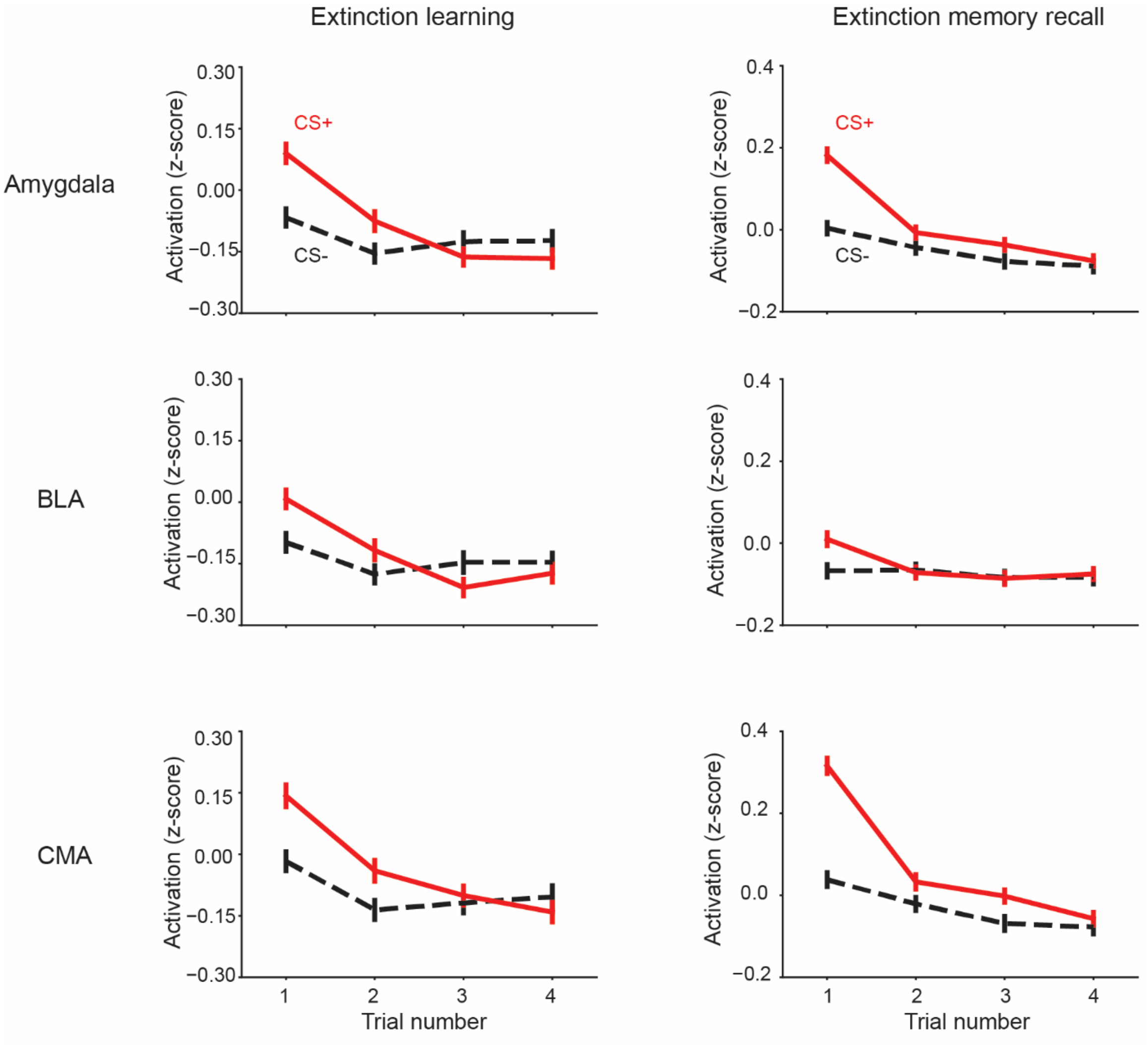

Fig. S4. Amygdala responses during extinction learning and extinction memory recall. During extinction learning (the first column), the amygdala responses were stronger to the CS+ relative to the CS- at the very beginning, then habituated quickly. Similar patterns were observed during extinction memory recall (the second column).

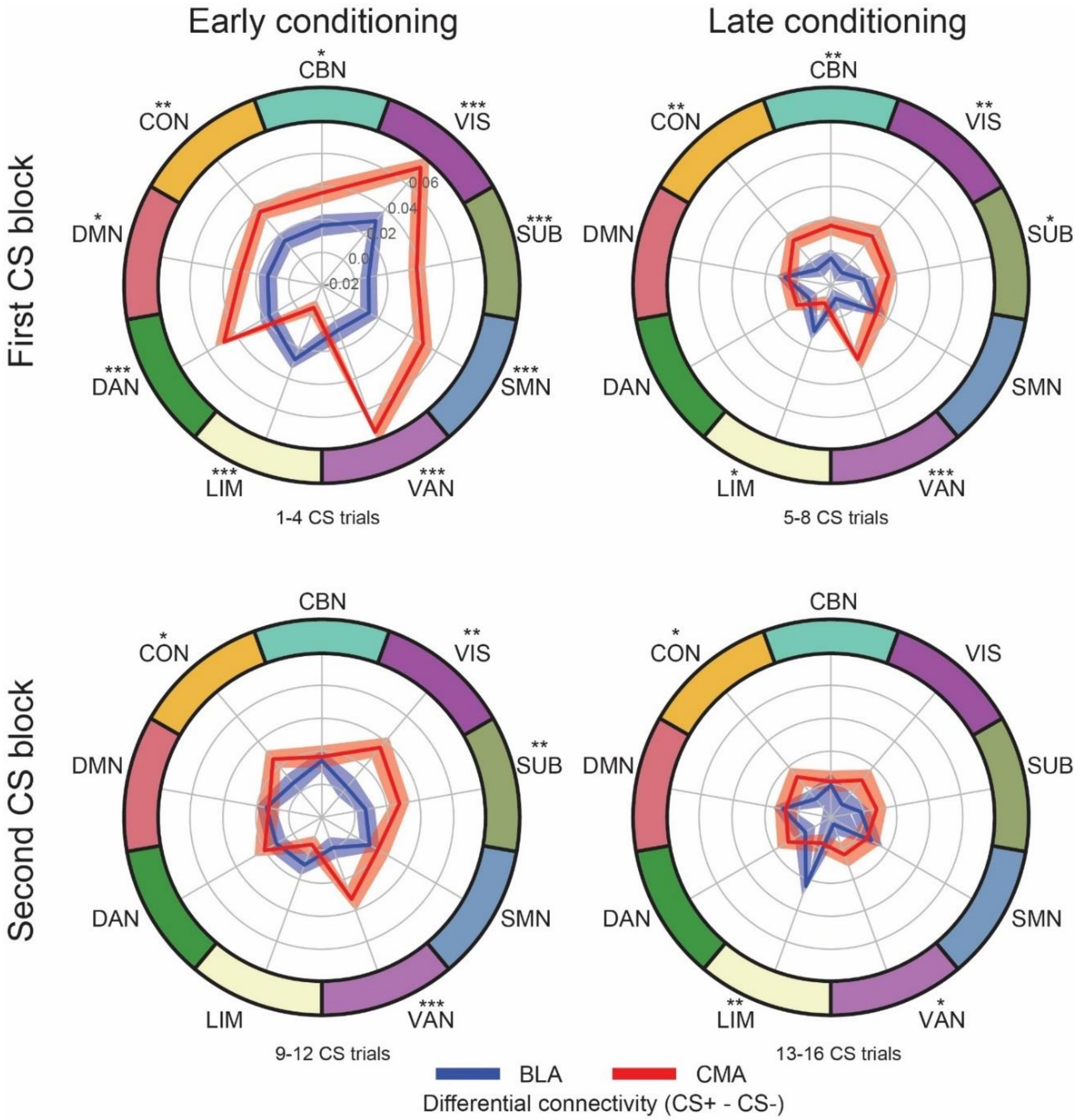

Fig. S5. Distinct functional connectivity patterns between the two amygdala subdivisions and distributed brain networks. The differential connectivity (connectivity during CS+ processing minus connectivity during CS- processing) between BLA/CMA and brain systems including the visual (VIS), subcortical (SUB), somatomotor (SMN), ventral attention (VAN), limbic (LIM), dorsal attention (DAN), default mode (DMN), frontoparietal control (CON), and cerebellum (CBN) networks, from early to late conditioning phase. Each panel represents connectivity estimated with 4 trials of each CS type. ***$P < 0.001$; **$P < 0.01$; *$P < 0.05$.

SI References

1. M. R. Milad, *et al.*, Neurobiological Basis of Failure to Recall Extinction Memory in Posttraumatic Stress Disorder. *Biological Psychiatry* **66**, 1075–1082 (2009).
2. M. R. Milad, *et al.*, Recall of Fear Extinction in Humans Activates the Ventromedial Prefrontal Cortex and Hippocampus in Concert. *Biol. Psychiatry* **62**, 446–454 (2007).
